# What Crisis? Competing Narratives of Mental Health in US Higher Education

**DOI:** 10.1007/s11013-026-09977-0

**Published:** 2026-03-07

**Authors:** Eugene Raikhel

**Affiliations:** Department of Comparative Human Development, University of Chicago, Chicago, United States

**Keywords:** College mental health, Crisis, Institutional capacity, Higher education, United States

## Abstract

The “mental health crisis” has become the dominant framework for understanding student distress in US higher education. Drawing on interviews with 35 mental health professionals working with college students, this paper examines how practitioners themselves understand and critique this crisis narrative. Rather than accepting or rejecting the crisis framing wholesale, professionals articulated five distinct and often competing ways of understanding what is happening in college mental health: (1) a mental health crisis emphasizing increasing student psychopathology; (2) a developmental crisis attributing distress to disrupted adolescent development from technology use and changing parenting practices; (3) an access crisis focusing on overwhelmed institutional capacity; (4) a crisis of meaning highlighting the semantic instability of diagnostic language as it circulates in vernacular usage; and (5) a crisis of higher education locating the problem in structural conditions and achievement culture. Each framing renders certain aspects of the situation visible while obscuring others, authorizes particular forms of expertise, and implies fundamentally different interventions. Building on critical scholarship that treats “crisis” as a framing device that performs political work, this paper argues that the multiplication of crisis narratives represents both a symptom of conceptual complexity and practitioners’ efforts to make visible what dominant framings obscure. Ultimately, professionals face the task of addressing patients’ needs while dealing with multiple simultaneous pressures and working with a limited capacity to address upstream causes of distress.

## Introduction

“Murray”^1^ is a clinical psychologist who directs the counseling center at a selective private university in the United States. I had asked to meet with him as part of a project on college mental health, and like many of the mental health professionals I spoke with, Murray’s training was eclectic, shaped by a range of distinct approaches and traditions in clinical psychology. When I asked Murray what the greatest challenges of his job were, he explained:

The overwhelming sense of responsibility for the lives of suicidal students I don’t know about. And the fear that all of the students who are quite comfortable sharing their suicidal ideation keep us very busy when they are really not suicidal in a literal sense. They’re suicidal in the sense of ‘I don’t want to live this way,’ but they don’t have a language for it. The pressure to meet the demands for students for clinical appointments on an individual basis because of this narrative of a mental health crisis… The easiest, top of the stress challenge is a sense of responsibility for these systemic pressures to meet demands, that are not taking into account the upstream reasons for, not only the students’ struggle, but the public narrative around how intense that struggle is and what it’s about.

Murray’s response suggests a professional caught at the intersection of multiple, simultaneous crises. There are individual students experiencing acute distress, some describing themselves as suicidal, who require immediate attention and care. There is the overwhelming demand for services that leaves Murray responsible for students he may never even meet. There is a semantic ambiguity: when students say they are “suicidal,” what exactly do they mean? 2 And underneath it all, Murray suggests, there are “upstream reasons” for student struggles that the dominant narrative of a “mental health crisis” obscures rather than illuminates. For Murray, the crisis is not simply one of student mental health, but of institutional capacity, semantic clarity, and misplaced causal narratives.

Murray was only one of a number of mental health professionals working with college students who spoke to me about their discontent with the current mainstream framing of the problem as a “crisis of college mental health.” But their critiques were not uniform. Rather, in questioning the dominant crisis narrative, these professionals articulated several alternative ways of understanding what is happening in college mental health, each of which foregrounds different problems, locates causality differently, and implies distinct interventions.

This paper examines how mental health professionals working with college students understand and critique competing crisis framings in college mental health. Drawing on interviews with 35 US mental health professionals (primarily counseling center staff and directors, as well as private practitioners who work extensively with students), I argue that “crisis” in college mental health is not a singular phenomenon but a set of multiple, competing framings that make different assumptions about the nature of the problem. I identify five such framings: the mental health crisis (emphasizing student psychopathology), the developmental crisis (emphasizing declining resilience), the access crisis (emphasizing institutional capacity), the crisis of meaning (emphasizing semantic instability), and the crisis of higher education (emphasizing structural causes).^3^ Each framing renders certain aspects visible while obscuring others, authorizes particular forms of expertise, and implies specific interventions while, in some cases, foreclosing alternatives.

This approach builds on critical scholarship that treats “crisis” not as a self-evident description of reality but as a framing device that performs political and conceptual work (Carr, 2019; Koselleck, 2006; Masco, 2017; Roitman, 2014). As Summerson Carr notes in her analysis of the “opioid crisis,” a shift in the object being qualified by “crisis” fundamentally changes the framing; the “opiate crisis” might be understood instead as a “pharma crisis” or a “poverty crisis” (2019: 165). Similarly, many mental health professionals ask whether the “student mental health crisis” is not perhaps better understood as an “access crisis” or a crisis of “overwhelmed services” (Anderson-Fye & Floersch, 2011), or indeed, as something else entirely. Each reframing directs attention to different causes, different responsible parties, and different potential solutions. In this sense, this paper engages with critical discussions of framing and problematization across the social sciences (Rabinow, 2003; Rosenberg, 1989).

Yet beyond simply mapping out these distinct framings, I argue that the multiplication of crisis framings tells us something important about the critical and practical work which professionals are undertaking. As Carr points out, “*crisis* is also a way of naming a problem which suggests that established conceptual apparatus is ill-equipped to make sense of what is going on and offer adequate answers and solutions,” (2019, p.163). I argue that professionals’ critical awareness of the tensions between multiple framings does not so much resolve them as it becomes part of the work they undertake. Ultimately, I argue that the crisis in college mental health might not be a single definable problem but rather that it points to our inability to articulate it in terms that allow for coherent responses. What we are confronting are multiple converging phenomena that resist integration within current conceptual frameworks. The proliferation of different crisis narratives serves as both evidence of this conceptual breakdown and an effort to overcome it.

My focus on mental health professionals as critical interpreters of their own field is deliberate. These professionals have what Holmes and Marcus (2005) call “para-ethnographic” perspectives on college mental health; they are not simply clinicians but also human scientists whose conceptual frameworks often approximate those of anthropology and who are “engaged in intellectual labors that resemble...our own methodological practices,” (595). Many of the professionals I interviewed conduct research on student mental health, theorize about cultural and social factors shaping student distress, and critically reflect on their institutions and their own professional practices. Many of their interests and concerns paralleled my own. This project thus treats them as “epistemic partners” rather than simply as interlocutors (Holmes & Marcus, 2005: 596).

This analysis contributes to a growing ethnographic literature on college mental health that has examined structural sources of distress, changing meanings of diagnostic labels, and practices surrounding psychiatric medications (Alsopp et al., 2023; Anderson-Fye & Floersch, 2011; Armstrong et al., 2023; Cifuentes et al., 2025; Cooper & McGee, 2017; Gordon, 2024; McKinney & Greenfield, 2010; Stanek & Mattson, 2024; Wiley, 2023; Wilson, 2025). It also extends anthropological analyses of crisis as a political and analytical category (Carr, 2019; Masco, 2017; Roitman, 2014) into the domain of institutional mental health care. Finally, by focusing on the vernacularization of diagnostic language and its effects, this paper contributes to longstanding anthropological and sociological discussions of how psychiatric categories circulate, transform, and shape both subjectivity and institutional practice (Brinkmann, 2016; Hacking, 1995, 2007; Horwitz & Wakefield, 2007; Kitanaka, 2012; Martin, 2007; Young, 1995).

Before turning to the crisis framings themselves, I first describe my methods and the mental health professionals who participated in this study.

## Methods

This paper draws on semi-structured interviews carried out in 2022 with 35 US mental health professionals working with college students, recruited through professional networks and snowball sampling.^4^ The majority (28) work or have worked in college counseling centers, while 7 are private practitioners; 11 are current or former counseling center directors. They represent diverse training backgrounds (clinical and counseling psychologists (46%), psychiatrists (29%), and clinical social workers (25%)) and work at institutions ranging geographically and by selectivity, though selective schools are overrepresented. Interviews were carried out by the author and explored professionals’ experiences, perspectives on rising help-seeking and diagnosis rates, and understandings of current challenges in college mental health. This study was approved by the Social and Behavioral Sciences IRB at the University of Chicago as study # IRB20-0190.

## A transformation in college mental health

Well before COVID-19, a crisis discourse pervaded college mental health (Anderson-Fye & Floersch, 2011; Kadison & DiGeronimo, 2005; Twenge et al., 2019). Studies showed steady increases in help-seeking and diagnoses over two decades, with treatment rates rising from 19 to 34% between 2007 and 2017 (Lipson et al., 2019). Surveys of US university presidents have, over recent years, found that a significant majority (as many as three-quarters of those surveyed in 2021) list the mental health of students as their most pressing issue (Melidona et al., 2021). This general sense of crisis, as well as the use of crisis language, has only been amplified by the pandemic and its aftermath. In subsequent years, the mental health of young people and college students has become an increasingly urgent topic in public discussions, with a range of important social actors, including the US Surgeon General, describing a crisis of youth mental health (Leshner & Scherer, 2021; Office of the Surgeon General, 2021).

Questions about increasing prevalence have recurred throughout college mental health’s history (Crook, 2020; Glass, 2024; Prescott, 2008), and yet when I asked professionals about what changes their field had undergone over the course of their careers, nearly all professionals working over 20 years described similar trajectories: starting when counseling centers focused on developmental issues—careers, relationships, identity—and experiencing a sea change toward managing acute psychiatric crises. As “Sara,” a director at a private selective university, explained, centers shifted from addressing ‘developmental needs’ to running ‘24/7 community mental health clinics.’

Despite this shift, it is important to note that the majority of college and university counseling centers do not offer inpatient treatment. Students who are deemed to need hospitalization are typically directed to a local hospital with which the center has an established relationship or, in the case of universities with medical schools, potentially an inpatient psychiatric unit at a teaching hospital. Beyond this, the scope of practice and organization of counseling centers at US higher education institutions varies widely, as does their level of funding. Some are integrated into broader student health centers, while others are standalone units offering some combination of talk therapy (often in a range of formats), medication management, outreach and psychoeducation, casework, assessment, peer education, and in some cases, specialized services for addictions, eating disorders, and other conditions. Increasingly, many centers contract with third-party online vendors which offer 24/7 therapy and other services through their digital platforms (Rosenbaum & Webb, 2024). The degree to which a particular center may offer all of these services varies widely, and centers also do a great deal of referring students to outside resources, including therapists in the community for longer-term psychotherapy and specialized services.

Many professionals described three key changes they had experienced over the course of their careers: overall increases in care-seeking (manifested in appointment volume), increases in severity of cases and symptoms, and significant uptick in anxiety relative to other diagnoses (Center for Collegiate Mental Health, 2019). As one director put it, “the volume is up, the intensity’s up.” Multiple professionals also echoed quantitative findings in describing a shift from depression to anxiety as the predominant presentation.

How did professionals explain these changes? Most emphasized complexity; “it’s multifactorial” was a common refrain. The most widely cited explanation for increased help-seeking was stigma reduction. Nearly everyone with over a decade’s experience noted changes in students’ willingness to discuss mental health openly. Others pointed to increased access for students with preexisting conditions, enabled by effective psychopharmaceuticals since the late 1980s and broader institutional efforts post-Americans with Disabilities Act. As one director explained, medications made it “much more possible for students with a significant mental health history, to be medicated, and to function well enough to go to college. Whereas before...they would have been in their parents’ attic,” (cf. Anderson-Fye & Floersch, 2011, pp. 501–502).

Yet beyond this, many articulated a range of arguments underlying which were competing ways of problematizing the fundamental issue of student mental health. In what follows, I examine each of these framings in detail, discussing their underlying assumptions about the problem, the assignment of responsibility, and potential solutions.

## Reframing the Crisis: Five Competing Narratives

The professionals I interviewed did not simply accept or reject the mental health crisis narrative. Rather, in articulating their discomfort with how the problem is currently framed, they offered alternative ways of understanding what is happening in college mental health. “Brian,” a counseling center director at a nonselective private university, exemplified professionals’ ambivalence: “I’m very skeptical of the crisis narrative… the media loves picking up on the crisis narrative.” Yet he acknowledged students were genuinely struggling. His skepticism questioned not whether problems existed but how we understand and name them.

What became clear across these interviews was that professionals were grappling with multiple simultaneous phenomena that the singular term “crisis” could not adequately capture. Their critiques coalesced around four alternative framings that stood in tension with, and sometimes in direct contradiction to, the dominant mental health crisis narrative. Each of these framings, including the dominant one, makes certain aspects of the situation visible while obscuring others, and each carries distinct implications for how institutions might respond.

### The Limits of the Pathology Narrative

The dominant framing—the idea of the mental health crisis—treats the problem primarily as one of increasing student psychopathology. This narrative, ubiquitous in public discourse and institutional discussions, highlights the quantitative increases in help-seeking, diagnoses, and symptom severity that professionals themselves described. It centers students’ subjective experiences of distress and provides a vocabulary for understanding those experiences.

This framing does important work. It validates students’ suffering as real and worthy of professional attention. It has helped reduce stigma by casting distress as illness rather than personal failure. Moreover, the framing of the mental health crisis connotes an urgency that demands immediate action. And indeed it has successfully mobilized institutional resources: universities have invested substantially in counseling services in response to the declared crisis (McCann & Cole, 2025).

Yet many of the professionals I spoke with expressed unease with how this framing individualizes what may be collective experiences and medicalizes what might be understood as reasonable responses to difficult social conditions. When Murray described students who say they are “suicidal in the sense of ‘I don’t want to live this way,’” he was pointing to something the pathology narrative struggles to accommodate: a form of distress that resists easy categorization as individual mental illness even as it demands serious attention and response. Additionally, a set of critiques of this framing of the problem has increasingly emerged in publications and presentations by counselors, counseling center directors, and mental health researchers (Bantjes et al., 2023; Glass, 2024; Gorman et al., 2024; Locke, 2025; Rosenbaum & Liebert, 2015; Xiao et al., 2017).

For example, longtime college mental health researcher and former director of the Center for Collegiate Mental Health at Penn State, Ben Locke, points out that while a “crisis” is meant to denote something acute and passing, the college mental health crisis has been continually invoked for some two decades, creating a “meaningless” sense of urgency without detail or nuance (2025). Significantly, Locke also argues that the crisis narrative is central in selling goods and services related to mental health: pharmaceuticals of course, but also increasingly digital tools and platforms (Locke, 2025; cf. Rosenbaum & Webb, 2024).

Others have disagreed specifically with the medicalizing assumptions underlying the mental health crisis framing (Rosenbaum & Liebert, 2015). Philip Rosenbaum and Heather Liebert, both psychodynamically oriented counseling center directors at small liberal arts colleges, argue that “framing current issues as a ‘mental health’ crisis can be problematic,” (180). Taking issue with the medicalized and diagnostic logic which accompanies the “mental health” framing, Rosenbaum and Liebert emphasize its unintended consequences. Foremost among these is the reduction of the counseling center’s role to the alleviation of symptoms, short-circuiting the possibility for students to make meaning of their experiences through therapy.

### A Crisis of Development: Technology, Parenting, and Resilience

Even as professionals debated whether the problem was one of student pathology, some articulated a framing that located the source of student distress in developmental and environmental changes: specifically, the effects of digital technologies and shifting parenting practices on young people’s capacity to handle difficulty. This framing, which circulates widely in public discourse about “coddled” or “fragile” youth (Lukianoff & Haidt, 2018), but also in recent arguments about the digital “rewiring” of childhood (Haidt, 2024), suggests that contemporary students arrive at college lacking emotional skills and coping capacities that previous generations developed through less structured, less protected childhoods.

“Molly,” a psychiatrist who had worked with college students for over two decades, put it bluntly: “It also is my experience in the last 10 years, I’ve just seen their levels of resilience go down, down, down, down, down. And my theory about that is, I want to blame it on the phones they have in their hands constantly, and this exposure to social media.” For Molly, the issue was not simply that students experienced more stress, but rather that they lacked the internal resources to manage that stress. “So what brings them in is something happens, or they worry something’s going to happen. And their capacity to cope with it is very limited.” The uncertainty inherent in college life becomes overwhelming for students who haven’t developed what another psychiatrist, “Melissa,” called the capacity “how to human.”

Melissa’s account linked technological and social changes in ways that many professionals echoed. Smartphones and social media, she suggested, had fundamentally altered how young people spend their time and attention. Rather than experiencing boredom, sitting with uncomfortable emotions, or engaging in unstructured social interaction (all experiences that might build emotional capacity), students had constant digital distraction available. “There’s a profound delayed sense of ‘how to human’ that I think is happening right now,” Melissa explained. “And it’s made worse by social media, which only glorifies black and white and... gold star stuff, and made worse by all of the technology that takes people out of their heads and out of time for reflection and discomfort.”

Brian described a shift from unsupervised childhoods that fostered resourcefulness to highly programmed ones where “parents are doing everything for you.” This, he argued, bred “a kind of helplessness”—students arrive at college never having developed the practical and emotional skills needed for independent living. As Levinson and McKinney point out, a parallel critique is sometimes applied to counseling itself: “Critics regard psy practices on campus as undermining the value of autonomy during a crucial period of transition to adulthood and as an extension of ‘helicopter parenting,’” (2013, p. 376).

The technology and resilience framing does several kinds of work simultaneously. Unlike the mental health crisis framing, it doesn’t locate the problem primarily in individual psychopathology requiring psychiatric treatment. Students aren’t necessarily mentally ill; they simply haven’t developed the necessary life skills. This framing redistributes responsibility: Parents bear some culpability for overprotecting their children, and technology companies might be implicated for creating addictive platforms. The implied solution is neither primarily clinical nor institutional but rather educational and skill-based: teaching coping strategies, promoting resilience, helping students develop the emotional and practical capabilities they lack.

Yet this framing also has its critics. “Jacob,” a counseling center director at a small selective liberal arts college, forcefully rejected what he saw as the victim-blaming implications of arguments about students lacking “resilience”—a term often invoked to suggest that contemporary students are somehow less capable of handling stress than previous generations:

“There’s something about lack of resilience, which is an attack on students...There’s no way students who are navigating social pressures, complexities of the world, multiple recessions, financial crashes, whatever, aren’t in some ways deeply resilient. You drop me as an 18-year-old into [this college]: I’d feel bad for myself!... And you know, meanwhile, at the socio-political level, where’s anything like resilience?”

From Jacob’s perspective, the resilience framing risks blaming young people for conditions they did not create—economic precarity, political instability, technological changes—while ignoring the question of whether social institutions themselves have become less resilient, less capable of providing the stability and support that development requires.

Moreover, several professionals acknowledged uncertainty about the technology and resilience narrative even as they invoked it. Research on social media’s effects remains contested (Orben, 2020; Twenge et al., 2018). Some seemed aware they were drawing on culturally available narratives, “kids these days” stories, rather than on settled scientific evidence.

Still, the technology and resilience framing had clear appeal for many professionals precisely because it offered an explanation for changes they felt certain they were observing: students who seemed less capable of managing ordinary stressors, less able to sit with discomfort, and more dependent on external structure and support.

### From Pathology to Capacity: The Access Crisis

For many professionals, the pressing problem was not so much whether students were getting sicker but whether institutions could respond to demand regardless of its source. This reframing, which can be called the access crisis, shifts the locus of concern from individual students to institutional capacity. “I think there’s an access crisis, but I’m not sure there’s a mental health crisis,” “Ross,” a counseling center director at a small liberal arts college, told me. His formulation does important work: It demands institutional resources and accountability without necessarily accepting that contemporary students are pathologically sicker than previous generations. Of course, from students’ perspectives this situation of “overwhelmed mental health services” translates to long wait times and difficulties accessing services when they are needed (Anderson-Fye & Floersch, 2011).

This framing makes visible what the mental health crisis narrative tends to obscure: the profound transformation of college counseling centers and the unsustainable conditions under which mental health professionals now work. Sara, who described her work shifting from “bread and butter” developmental counseling to running “community mental health clinics,” was articulating this crisis of institutional capacity. Her account highlighted not only increased volume but also a fundamental change in the nature of the work: from supporting students through normative developmental challenges to managing acute psychiatric crises on an ongoing basis.

The access crisis framing draws attention to the labor conditions of mental health professionals themselves. Several directors I spoke with described pervasive burnout among their staff, high turnover rates, and persistent difficulty filling vacant positions, all phenomena which have been documented in the literature. Counseling center staff report pervasive burnout (88% of directors, 92% of clinicians), high turnover, difficulty recruiting, and lower salaries than comparable settings, leading many to leave for private practice (Gorman et al., 2022, 2024; Walden et al., 2022). As one psychiatrist at a large state university put it, after explaining to me that she had just put in her two weeks’ notice earlier that day, “[I]t still feels kind of like abusive to have… highly educated clinicians who are not paid anything, who are constantly, day in day out, talking to people about their deep trauma, and simultaneously hearing around the university… ‘You’re not doing enough, you could be doing more.’” Current and former counseling center directors, Philip Rosenbaum and Richard Webb have similarly argued that such dynamics leave college counselors “…feeling that our role within the college community is neither appreciated nor truly understood. This creates a significant crisis in our sense of identity and leads to feelings of moral injury,” (2023).

A focus on capacity also suggests a distinct set of interventions, and a number of actors in the college mental health space have followed on this framing by developing new metrics and arguing for a reconceptualization of counseling centers’ objectives. For example, a team at the Center for Collegiate Mental Health (CCMH) at Penn State developed a new metric called the Clinical Load Index (CLI) to help support decisions about staffing (Locke, et al., 2024). More recently, two reports spearheaded by the Association for University and College Counseling Center Directors (AUCCCD) have made the argument that asking, “how to meet clinical demand” is an “outdated and misleading” question, instead suggesting that institutions ask, “what demand are we going to meet? And what resources do we need to do that?” (Gorman et al., 2024, p. 5; Glass et al., 2025). As a response to the access crisis, these reports encourage institutions of higher education to stop attempting to do everything at once and instead organize their scope of practice in a more deliberate and carefully considered way.

“We know that something’s happening upstream,” one counseling center director explained, “whereby we are deluged with students requesting our services downstream. And so, we’ve come to think of it as like we’re a symptom of some sort of larger systematic issue.” This last formulation is particularly significant because it suggests that the access crisis itself may be symptomatic and that understanding why services are overwhelmed requires looking beyond simple increases in student pathology. This opens toward the other alternative framings that professionals articulated, each of which highlights different aspects of how we understand the problem.

### The Instability of Meaning

Perhaps the most conceptually sophisticated critique offered by professionals concerned not whether students were sick or whether services were adequate, but whether we even know what we’re talking about when we discuss student mental health. This framing, which I call the crisis of meaning, emerged most clearly in conversations with several counseling center directors who had noticed something puzzling: students seemed to be using mental health and diagnostic language in ways that didn’t quite align with clinical definitions, creating a kind of semantic instability that affected both practice and research.

Ross was one of several professionals who articulated this concern. He described students using diagnostic terms like “panic attack” or “social anxiety” to describe what he understood as “kind of a fairly normal nervousness.” The problem, he suggested, was not students’ use of this language per se; after all, they were drawing on available cultural vocabularies to communicate distress. Rather, the difficulty arose when researchers and clinicians interpreted students’ vernacular usage as if it corresponded to diagnostic criteria. Students might describe themselves as having “anxiety” in a colloquial sense (feeling worried, stressed, or nervous) while surveys and studies counted these self-reports as evidence of anxiety disorders. This created what Ross called an “upcoding” effect, artificially inflating apparent rates of psychopathology.

“Robert,” a counseling center director at another selective liberal arts college, framed the issue more broadly and conceptually:

“Diagnostic language is at once a helpful pathway to...expression of these inner states, and at the same time...a limiting pathway that precludes other things and other possibilities...I think that’s why people are using the language so much, actually, because it’s giving them a socially legitimate way to fall into this cultural container that’s opening up. But...if everybody is using that language, what is the value of the language, right, and does it mean what we think it means?...Because if we all have disorders, they aren’t disorders, right? It’s just life.”

Robert’s formulation captures a paradox at the heart of contemporary college mental health. On one hand, the proliferation of mental health language represents progress; it provides students with vocabulary for articulating distress, offers validation for suffering, and creates “socially legitimate” ways to seek help and access accommodations. The reduction in stigma that many professionals celebrated has been accomplished partly through making mental health concepts widely available and articulable. On the other hand, as diagnostic categories circulate beyond clinical contexts and take on vernacular meanings, they undergo semantic transformation. The very ubiquity that makes them useful also destabilizes their clinical specificity. If everyone describes themselves as “anxious” or “depressed,” these terms begin to function more as general idioms of distress than as markers of specific psychiatric conditions (Nichter, 2010).

This vernacularization creates what might be understood as a looping effect, borrowing Ian Hacking’s concept of how human kinds are “moving targets,” (2007). Students learn to interpret their experiences through available diagnostic categories. These interpretations become part of their experience, shaping how they understand themselves and how they explain their feelings to others. The categories themselves shift in meaning through this process of circulation and reinterpretation. When researchers then use these same categories to study mental health, assuming semantic stability, they may be measuring something quite different from what they intend. Data from the CCMH suggested exactly this problem: Students appeared to be using “mental health” as “a general expression of distress” rather than a specific clinical category, making it unclear what self-report measures were actually capturing (Center for Collegiate Mental Health, 2021).

The crisis of meaning also manifests in institutional dynamics. As Gorman and colleagues documented, when students broadly invoke “mental health” in conversations with faculty and staff, these institutional actors often become “more heightened and reactive,” immediately referring students to counseling services for what might be relatively minor or transient distress (2024). Faculty members, uncertain about the severity implied by students’ mental health disclosures and anxious about potential liability, default to professional referral. This contributes directly to the access crisis, but it does so through a mechanism of semantic transformation rather than through actual increases in severe psychopathology. The problem is not only or even primarily that students are sicker, but that the language of mental health has become the default vocabulary for communicating any form of struggle or difficulty.

This framing resonates with a range of critical scholarship on mental health and illness, both inside and outside of higher education. For example, a range of scholars have pointed to the expansion and loosening of diagnostic categories, as well as the increasing use of broad idioms such as “distress” and “trauma,” and suggested these may themselves be linked to trends such as overdiagnosis in psychiatry, increasing cultural sensitivity to harm, and the rise of the wellness industries (Brinkmann, 2016; Fassin & Rechtman, 2009; Haslam, 2016; Rose, 2006). Anthropologists and sociologists have demonstrated the multiple ways in which diagnoses in higher education have not simply been carriers of stigma but have often come to be the objects of meaning making, identity formation, social capital, and the key to access various forms of treatment, care, and accommodation (Armstrong et al., 2023; Stanek & Mattson, 2024).

Some professionals described even more explicit forms of identification with diagnostic categories. “Olivia,” a therapist at a small liberal arts school, told me about a TikTok trend she’d observed where “people are identifying with having dissociative identity disorder, because they’re identifying different emotions as different alters. So instead of...’I feel sad right now,’ it’s this sense of, I must have another alter.” What struck Olivia was not that students were deliberately faking symptoms but rather that they were genuinely interpreting ordinary emotional experiences through a diagnostic framework that transformed those experiences into evidence of pathology. Diagnostic categories, in Armstrong and colleagues’ apt phrase, had become “campus technologies,” resources that students deployed strategically to make sense of experience, access accommodations, and navigate institutional demands, but which in the process took on meanings quite distant from their clinical origins (2023).

While highlighting semantic complexity, this framing risks being weaponized to dismiss student experiences—to suggest students are “merely” using diagnostic language and do not have real problems. This concern surfaced in several webinars critiquing the mental health crisis framing, in which participants were at pains to emphasize that they were in no way meaning to disregard students’ suffering.

### Structural Conditions and Systemic Causes

The fifth framing that emerged from my interviews represented the most radical departure from the mental health crisis narrative. Rather than locating the problem in individual pathology, developmental rupture, institutional capacity, or semantic instability, this framing identified the source of student distress in the structure and culture of higher education itself, and indeed, in broader social and economic conditions. If we must speak of a crisis, several professionals suggested, it is not fundamentally a mental health crisis at all but rather a crisis of higher education and of the social conditions in which young people are coming of age.

This framing makes visible what the mental health crisis narrative most thoroughly obscures: the “upstream reasons” that Murray referenced, the systemic pressures and structural conditions that may be producing distress in the first place. “John,” who had recently retired after directing a flagship public university’s counseling center, articulated this perspective when he reflected on changes over his long career:

I mean, you have to look at the broader context...of what’s been happening in society for all of us, you know, in the level of stress that people are under now, compared to perhaps 20 years ago...economically, politically, shifts in so many things. And with stress comes more pathology, I mean, all of us act crazy, you know, if we’re under enough stress.

John went on to describe what he saw as a fundamental shift in the culture of higher education itself: a movement toward what Byung-Chul Han has called “an achievement society,” characterized by an intensification of pressure and competition that affected students’ relationship to their own education and to themselves (2015). “The pressure to do well, and the pressure to get As and the pressure to succeed and your identity being wrapped up in academic success,” John explained, describing students “groomed from high school to get straight As...SAT scores, to be in five different clubs, to be the perfect candidate for Harvard.” This professionalization of studenthood means that students approach college not as a time for exploration and development but as another credential to be optimized, another competition to be won (Martin, 2022). Others emphasized the ubiquity of “grind culture” (Wiley, 2023) or pointed to other dysfunctional aspects of “campus culture.” Such arguments resonate with recent ethnographic work, such as Gracie Wilson’s finding that students at a liberal arts college valorized stress and treated it as a form of social capital, a way to demonstrate commitment and a way to signal moral worth (2025).

In a recent publication, longtime counseling center director, Gary Glass, summed up this framework, writing “The future of college mental health may require articulating the actual issue as a higher education crisis, exposing parallels between the mental state of students and the mental state of higher education.” Glass argues that college students and the institutions they attend are caught in the same bind: obsessed with “achievement” and “excellence,” defining success entirely in terms of rankings and narrow metrics, and focused on “narratives of an enriching college experience that provides access (and promises) to structures of security, if not empowering wealth” (Glass, 2024, pp.795–796).

Other professionals I spoke to pointed to economic precarity as a source of distress that individual therapy could do little to address. “Carol,” a psychiatrist at a large public university, emphasized that contemporary college populations include many students facing genuine material hardship: “Our colleges now consist of people who’ve lived through significant hardship...who deal with day-to-day discrimination, and who don’t have enough money to get by, who sometimes don’t have enough food to get by. And you don’t have your basic needs met, and that’s a big risk factor for anxiety and depression and start of any other illness as well.” For these students, anxiety and depression might be less symptoms of individual psychopathology than reasonable responses to genuinely precarious circumstances.

Several professionals also noted that students themselves were increasingly gesturing toward structural analyses of their own distress. Murray described being surprised by how often students had begun “introducing capitalism” into therapy conversations over the past several years. “They are connecting the dots between their psychological struggles and systemic reality,” he explained. Some students used “capitalism” to reference the pressure to constantly achieve and produce—“I have to do all of these things, or I won’t be successful.” Others deployed it to name a sense of exploitation—“the exhaustion that I feel is because this world is exploiting me, this university needs my money.” We can imagine how these distinct articulations of “capitalism” may map onto different social trajectories and even distinct institutions with their varying demographic profiles. For many students at selective institutions, invoking capitalism may mean articulating a critique of what might be called the logic of constant optimization and performance, whereas at large public universities and less selective institutions, students’ invocations of capitalism may reference direct experiences of economic exploitation and precarity. The use of this terminology also suggests that students are actively drawing upon concepts they have encountered in their classes and readings. Overall these students seemed to be using “capitalism” less as an analytic term than as a kind of idiom of distress, albeit one which eschewed the individualization inherent in the mental health crisis framing, pointing instead to the ways their distress was connected to larger social and economic systems (Nichter, 2010). And yet this also points to the bind which counselors find themselves in, as they experience a tension between validating some students’ structural interpretations of their distress and continuing to operate within a therapeutic framework which remains fundamentally individualized.

## Five Crises, Distinct Framings

These five ways of understanding the crisis in college mental health entailed a range of distinct perspectives, some of which were integrated or braided into specific accounts, but others of which contained seemingly irreducible tensions with one another. None of the mental health professionals I spoke with focused on only one of these crisis narratives; all of them drew on multiple framings to understand the situation.

At the same time, each of these framings represents a relatively distinct way of understanding what kind of problem we face. The mental health crisis framing treats distress as located primarily in individual students’ minds and brains, requiring clinical intervention. The crisis of development framing understands it as a symptom of the disruption of normative developmental processes by new technologies and parenting styles. The access crisis framing sees an institutional problem of capacity and resource allocation. The crisis of meaning framing highlights an epistemic problem about the stability and meaning of categories. The crisis of higher education framing treats the problem as a social and structural one requiring systemic rather than individual change.

Significantly, some of these frameworks seemed more compatible with some others, while in some cases, there seemed to be particularly strong tensions. As I have mentioned, the access crisis framing was often braided together with references to semantic uncertainty or structural conditions, in part because it begged the question of what created the increases in demand in the first place. Of all the frameworks, the crisis of development was one which was most likely to be articulated on its own, and even to be argued against by other professionals. For example, the psychiatrist Melissa, who spoke about learning “how to human,” did not mention changes in the meaning of diagnostic terms or structural factors, while most of those who focused on the latter two frameworks did not mention the crisis of development. When they did, it was, like Jacob, to reject this framing as an “attack on students.” This is related to the way in which the crisis of development framing has to some degree become coded as centrist or center-right in today’s rather polarized political atmosphere, whereas an emphasis on structural issues is coded politically as left.

Despite these differences, each of these framings highlighted aspects of professionals’ experiences and observations, and each captures something real about the contemporary landscape of college mental health. They make competing claims about where to look for causes, who bears responsibility, what counts as evidence, and what kinds of interventions might be appropriate. Professionals must somehow navigate the factors and issues raised by all five framings at once in their daily work: responding to individual students in crisis while managing institutional constraints, uncertain about what diagnostic language means, and aware that their interventions cannot address the structural conditions producing distress in the first place. It is this impossible navigation, perhaps, that constitutes the greatest challenge that college mental health professionals face.

## Discursive Work and Its Limits

Anthropologists have shown that “crisis” declarations do political work (Carr, 2019; Koselleck, 2006; Roitman, 2014). Koselleck notes that “crisis” carries urgency from its Greek etymology—an inflection point requiring decision. This urgency can foreclose critical questioning about the problem’s nature (Masco, 2017; Roitman, 2014). As Joseph Masco has put it, “In our moment, crisis blocks thought by evoking the need for an emergency response to the potential loss of a status quo, emphasizing urgency and restoration…,” (2017, p. S73). And yet, college mental health demonstrates that crisis declarations can also become sites of contestation. When professionals question whether we have a “mental health crisis” versus an “access crisis” or “crisis of higher education,” they engage in important discursive work: challenging causal narratives, redistributing responsibility, arguing for different interventions. This multiplication of crisis framings represents efforts to make visible what dominant framings obscure.

Yet the relationship between this discursive work and material or political effects remains complex and uncertain. There is some evidence of efforts to translate these critiques into institutional action. As documented earlier in this paper, a number of counseling center professionals have published widely in both academic and professional venues, developed new metrics like the Clinical Load Index to guide resource allocation, and produced reports through the Association for University and College Counseling Center Directors that explicitly challenge the dominant crisis framing (Glass et al., 2025; Gorman et al., 2024; Locke et al., 2024). These publications argue that institutions should reconceptualize their approach, asking not “how to meet all clinical demand” but “what demand are we going to meet?,” and advocate for more realistic scopes of practice, distributed models of student well-being, and recognition that counseling centers cannot and should not attempt to be “everything to everyone,” (Gorman et al., 2024: 5).

These efforts represent serious attempts to reshape institutional responses. The AUCCCD reports in particular aim to influence decision-makers at colleges and universities by providing alternative frameworks and actionable recommendations (Glass et al., 2025; Gorman et al., 2024). Some professionals I interviewed described presenting these arguments to their campus leadership, using the language of access crisis or structural conditions to advocate for changes beyond simply hiring more counselors. Whether and how these efforts will translate into actual institutional changes remains to be seen. The mental health crisis framing continues to dominate public discourse and many institutional responses precisely because it is most legible to decision-makers and most compatible with existing structures of expertise and intervention. Universities have indeed invested substantially in counseling services, but largely by expanding clinical capacity rather than fundamentally reconceptualizing their approach or addressing the structural conditions that professionals identify as sources of distress.

The alternative framings articulated by professionals may face significant obstacles to institutional uptake for precisely the reasons they are analytically important. The crisis of meaning unsettles the very categories through which institutions measure and respond to student distress. The crisis of higher education implicates the institutions that employ mental health professionals in producing the problems they are tasked with solving. These framings ask uncomfortable questions that decision-makers may prefer not to confront. Moreover, mental health professionals themselves, however, thoughtful their critiques, operate within institutional constraints that limit their capacity to act on their analyses. A counseling center director who recognizes that achievement culture produces student distress cannot unilaterally transform that culture, even if they articulate a compelling critique of it.

There is another, perhaps more sobering interpretation of the multiplication of crisis framings documented in this paper. Rather than representing productive contestation or nascent political mobilization, the proliferation of competing frameworks may instead reflect the fundamental impossibility of the therapeutic task as currently constituted. Mental health professionals encounter student suffering that results from the convergence of developmental, technological, economic, cultural, and institutional factors that no individual clinician or counseling center can possibly address comprehensively. The welter of competing frameworks may be less a set of alternative solutions than a symptom of professionals grappling with an impossible demand: to provide individualized therapeutic interventions for what are fundamentally collective and structural problems.

From this perspective, the competing crisis framings represent different attempts to make sense of this impossibility, each highlighting certain aspects of an intractable situation while necessarily bracketing others. Professionals articulate these alternative framings not necessarily because they believe they can act on them, but because the dominant mental health crisis narrative feels inadequate to capture their experience. In a way that echoes the findings of other anthropologists working with psy professionals, the proliferation of framings may reflect ethical and intellectual struggle more than political strategy, an effort to comprehend a situation that exceeds available frameworks rather than a coordinated challenge to dominant narratives (Brodwin, 2013; Weiner, 2026).

## The Crisis for Professionals

Perhaps the deepest crisis, then, is the one professionals themselves experience: being tasked with responding simultaneously to multiple factors and conditions, some of which are in tension with one another. They must address individual students in acute distress while managing institutional constraints and overwhelming demand, uncertain about what students’ diagnostic language actually means, and aware their interventions cannot address structural conditions producing distress.

Murray captured this precisely. His “overwhelming sense of responsibility” derives not from any single aspect of his work but from the simultaneity of contradictory demands: individual students needing immediate help, services overwhelmed by volume, language that doesn’t quite capture what students mean, and upstream causes individual therapy cannot address. The responsibility he describes is not only personal but structural, a consequence of being tasked with solving problems exceeding mental health services’ capacity.

Several professionals described what might be called moral distress—arising when one knows what should be done ethically but constraints make it impossible. They know many students need help they cannot provide. They know the help they can provide may not address root causes. They know their institutions contribute to stress they’re tasked with helping students manage. And they know students most in need may be overlooked in overwhelmed systems.

The professionals in this study demonstrate that critical awareness of these tensions does not resolve them but becomes part of the work itself. They must provide care while questioning the frameworks within which care is offered. They must respond to immediate needs while advocating for upstream changes. They must work within institutions while recognizing those institutions’ role in producing the problems they’re addressing. A few professionals I spoke to articulated this dilemma explicitly. For instance, Brian described the “business model” of the university as “creating a monster that’s feeding itself,” and added, “I mean, I sometimes feel guilty for working here, because we’re latching so many students with student loan debt that they have no hope of ever repaying.” More pointedly, when I asked Jacob about how he would explain increasing rates of help-seeking and acuity among students, he highlighted the professionalization of studenthood, but added:

And I often get the impression that we’ve played a role in this. We promote ourselves in this, we see ourselves as offering something really important at the end of the rainbow. Like we sort of have to as well, right? Like, if you’re going to go through all of that to get here, to get to college, it better be worthwhile. And I don’t think there’s any way that we can deliver [laughs] on what the expectation is. And we really don’t like that feeling, that we can’t deliver, because nobody fucking could, right? … And then students get disappointed, and they’ve had so much performance that they’ve had to do for so long. This is now the first place where they actually have to deal with that disappointment.… [W]e don’t want to be seen as the bad object. And yet, I think increasingly, that’s the important role of college. We’re the place that’s going to disappoint you.

Rather than pointing the finger at the “upper administration,” he situates himself, and by extension, the counseling center, as complicit in creating a system that produces this particular vision of college. This is perhaps less because he is himself committed to such a vision than because of his understanding of the structural role played by the therapist in the college counseling center. He is both a therapist and a symbolic representative of the university—the one onto whom frustrations about the institution are likely to be projected—and indeed, whose job it is, in his view, to create a kind of holding space for these feelings of disappointment and anger.

## Consequences for Research and Practice

Of the frameworks reviewed here, the crisis of meaning, in particular, raises urgent questions for mental health research. If students use diagnostic language in ways differing from clinical definitions, what do large-scale surveys measure? The CCMH’s finding that students use “mental health” as a “general expression of distress” rather than a specific clinical category suggests research claiming to document increases in diagnosable conditions may actually document something else—perhaps increased comfort with mental health vocabulary, shifting cultural idioms for expressing distress, or changing expectations about what warrants professional help (Center for Collegiate Mental Health, 2021).

This doesn’t mean students aren’t genuinely suffering or that distress increases aren’t real. Rather, it demands more sophisticated approaches to understanding self-report data. When students say they experience “anxiety” or “depression,” we cannot assume they’re using these terms corresponding to diagnostic criteria (cf. Armstrong et al., 2023). Increases in such self-reports may reflect increases in clinically significant psychopathology or shifts in how people interpret and label experiences.

For practice, recognizing multiple crisis framings suggests institutions cannot “solve” the crisis without deciding which crisis they’re addressing, and recognizing different crises require different responses. If the problem is primarily psychopathology, then expanded clinical services are appropriate. If it’s institutional capacity, we need not only resources but different service models and clearer scope boundaries. If it’s semantic instability, we need better mental health literacy and recognition that how people talk about mental health shapes institutional demand. If it’s structural, we need institutional changes addressing achievement pressure, economic precarity, and conditions producing distress.

In practice, all are likely needed to some degree. But the mental health crisis framing’s dominance has meant responses are overwhelmingly clinical without adequate attention to other dimensions. Several professionals suggested what’s needed is not simply more mental health services but distributed responsibility for student well-being across campus, with faculty, residential life staff, and peer networks addressing certain forms of struggle, and institutions examining their own cultures and practices contributing to distress.

## Conclusion

Many of my interviewees felt that the current welter of social, technological, and economic changes buffeting young people profoundly resisted explanation through our current concepts and frameworks. As Summerson Carr suggests, some used the language of crisis to suggest that our “established conceptual apparatus is ill-equipped to make sense of what is going on,” while others were profoundly skeptical of crisis framings (2019, p.163). Gesturing toward the effects of “screens and technology and parenting styles and the economy” on young people’s mental health, Brian suggested, “I would love to be able to read the perspective 20 years from now that looks back and provides a different kind of contextualization of this with a clearer view of you know, everything because I feel like we sort of are in the middle of something that we don’t really even fully grasp.”

Brian’s comment points to genuine epistemic humility in the face of rapid social, technological, and cultural change. But his wish for future clarity also suggests something important about the present: we are not simply dealing with a technical problem that better data or clearer understanding would resolve. The multiple crisis framings in college mental health reflect deep tensions in how we understand the relationship between individual suffering and social structure, between personal responsibility and institutional obligation, between medical authority and lived experience, between therapeutic intervention and political change. These tensions cannot be resolved through better research or more resources, though both would help. They are, instead, constitutive of contemporary approaches to mental health in institutional settings.

Mental health professionals must navigate these tensions daily without the luxury of theoretical resolution. They must respond to individual crises while recognizing structural causes they cannot address. They must use diagnostic language while questioning its stability and meaning. They must work within medical frameworks while understanding their limitations. They must provide care while acknowledging that the scope of what clinical intervention can accomplish is narrow relative to the breadth of factors producing student distress.

This paper has documented five competing crisis framings not to adjudicate between them or to propose a synthesis, but rather to make visible the conceptual and practical predicament that mental health professionals face. Their articulation of alternative framings represents important intellectual work: rigorous attempts to understand a complex and rapidly changing situation. Whether this discursive work will translate into institutional changes, policy shifts, or altered practices remains an open question. What seems clear is that the dominant mental health crisis framing, despite its institutional success in mobilizing resources, cannot adequately capture the full scope of what is happening in college mental health or address the multiple sources of student distress that professionals identify.

If there is value in documenting these competing framings, it lies not in the idea that articulating alternatives will automatically transform practice, but rather in making explicit the contradictions and impossibilities that structure mental health work in higher education. Murray’s answer to my question about his greatest challenges was not an isolated complaint but a nuanced diagnosis of the multiple demands structuring college mental health work, demands that often pull in contradictory directions and that frequently exceed what counseling services can possibly address. His analysis, like those of the other professionals I interviewed, deserves to be taken seriously not because it points toward ready solutions, but because it illuminates the predicament of attempting to provide individualized therapeutic care for suffering that has collective and structural origins. Whether institutions and policymakers are prepared to confront this predicament, to recognize the limits of clinical intervention, to address the upstream causes of student distress, to transform the conditions that produce suffering rather than simply expanding services to manage its symptoms, remains to be seen.

## Competing interest

The authors declare no competing interests.

## Data Availability

The interview data generated for this study are not available to be shared for reasons of confidentiality.

## Footnotes

1 All of the names of interview participants in this paper are pseudonyms. Potentially identifying details about their careers or institutions have also been concealed in order to maintain anonymity.

2 It is important to note that throughout the rest of our conversation Murray made it very clear that he was not dismissing the concerns of the students who came to him for help with their suicidal ideation. While acknowledging their distress as very real, Murray suggested that some of these students were using the language of suicidal ideation as a kind of idiom of distress, a culturally validated mode of communicating problems which may not necessarily be psychopathological (Nichter, 2010). Thanks to Anna Mueller for emphasizing this point.

3 Of course, these are all ways in which “crisis” is applied to mental health in higher education as a collective condition. “Crisis” is also often invoked to refer to the individual acute psychological states of particular students. While this is beyond the scope of this paper, it is worth noting that on this individual psychological level, “crisis” is not always negative: Erik Erikson’s notion of “identity crisis” suggests crisis as a necessary part of the developmental process, valorizing it as productive (1968). Thanks to Eileen Anderson for pointing this out.

4 Mental health professionals were recruited through a range of methods. Recruitment was open to any mental health professionals primarily working with college students in the United States. An initial recruitment email to existing colleagues and acquaintances yielded approximately 16 contacts who were interviewed. Each interviewee was asked to recommend additional contacts, a method which yielded an additional 12 interviews. Finally, an additional 35 potential interviewees, particularly current and former counseling center directors, were contacted directly, of whom 7 responded and agreed to be interviewed. These were chosen in an effort to include interviewees working at a range of different types of higher education institutions, in terms of public/private status, selectivity, and size of student population.
